# Flow cytometric analysis and microsatellite genotyping reveal extensive DNA content variation in *Trypanosoma cruzi* populations and expose contrasts between natural and experimental hybrids

**DOI:** 10.1016/j.ijpara.2009.04.001

**Published:** 2009-10

**Authors:** Michael D. Lewis, Martin S. Llewellyn, Michael W. Gaunt, Matthew Yeo, Hernán J. Carrasco, Michael A. Miles

**Affiliations:** a Pathogen Molecular Biology Unit, Department of Infectious and Tropical Diseases, London School of Hygiene and Tropical Medicine, Keppel Street, London, WC1E 7HT, UK; b Instituto de Medicina Tropical, Facultad de Medicina, Universidad Central de Venezuela, Caracas, Venezuela

**Keywords:** *Trypanosoma cruzi*, Hybridisation, DNA content, Ploidy, Microsatellite, Genetic exchange

## Abstract

*Trypanosoma cruzi* exhibits remarkable genetic heterogeneity. This is evident at the nucleotide level but also structurally, in the form of karyotypic variation and DNA content differences between strains. Although natural populations of *T. cruzi* are predominantly clonal, hybrid lineages (TcIId and TcIIe) have been identified and hybridisation has been demonstrated in vitro, raising the possibility that genetic exchange may continue to shape the evolution of this pathogen. The mechanism of genetic exchange identified in the laboratory is unusual, apparently involving fusion of diploid parents followed by genome erosion. We investigated DNA content diversity in natural populations of *T. cruzi* in the context of its genetic subdivisions by using flow cytometric analysis and multilocus microsatellite genotyping to determine the relative DNA content and estimate the ploidy of 54 cloned isolates. The maximum difference observed was 47.5% between strain Tu18 cl2 (TcIIb) and strain C8 cl1 (TcI), which we estimated to be equivalent to ∼73 Mb of DNA. Large DNA content differences were identified within and between discrete typing units (DTUs). In particular, the mean DNA content of TcI strains was significantly less than that for TcII strains (*P* < 0.001). Comparisons of hybrid DTUs TcIId/IIe with corresponding parental DTUs TcIIb/IIc indicated that natural hybrids are predominantly diploid. We also measured the relative DNA content of six in vitro-generated TcI hybrid clones and their parents. In contrast to TcIId/IIe hybrid strains these experimental hybrids comprised populations of sub-tetraploid organisms with mean DNA contents 1.65–1.72 times higher than the parental organisms. The DNA contents of both parents and hybrids were shown to be relatively stable after passage through a mammalian host, heat shock or nutritional stress. The results are discussed in the context of hybridisation mechanisms in both natural and in vitro settings.

## 1 Introduction

Chagas disease is estimated to cause ∼13,000 deaths per year and the loss of 649,000 disability adjusted life years (DALYs) (WHO, 2002); it is considered to be a neglected tropical disease (Hotez et al., 2006). The aetiological agent, *Trypanosoma cruzi*, is harboured by at least 10 million people and is a zoonosis endemic across the vast majority of Latin America and into the southern states of the USA (Schofield et al., 2006). *Trypanosoma cruzi* is genetically highly diverse: multilocus genotyping consistently reveals six distinct ‘discrete typing units’ (DTUs), which have been divided into two ‘major subdivisions’ termed TcI and TcII; TcII being further split into five DTUs: TcIIa-e (Brisse et al., 2000, 2001). The individuality of each DTU is maintained by independent, predominantly clonal evolution resulting in highly distinctive genotypes (Tibayrenc et al., 1993; Oliveira et al., 1998; Barnabé et al., 2000; Brisse et al., 2000). *Trypanosoma cruzi* is considered to be diploid (Gibson and Miles, 1986; Tibayrenc et al., 1986), but there is evidence that aneuploidy may be a feature of some strains (Obado et al., 2005; Branche et al., 2006). Moreover, dramatic differences in DNA content between strains have been documented (Dvorak et al., 1982), yet the causes and consequences of this phenomenon remain poorly understood.

*Trypanosoma cruzi* has been considered the paradigm for clonal evolution in parasitic protozoa (Tibayrenc and Ayala, 2002). Nevertheless, the identification of strains with recombinant genotypes (Machado and Ayala, 2001; Brisse et al., 2003) has made it clear that recombination, though rare, has had important consequences for the evolution of *T. cruzi*. A large amount of data now supports the views, firstly, that TcI and TcIIb are ancient lineages, and secondly, that two *T. cruzi* DTUs (TcIId and TcIIe) are the products of one or more hybridisation events between a TcIIb parent and a TcIIc parent (Machado and Ayala, 2001; Brisse et al., 2003; Westenberger et al., 2005; de Freitas et al., 2006). The evolution of TcIIa and TcIIc is inadequately understood at present; some data have indicated that these DTUs may also have a common, hybrid origin (Sturm et al., 2003; Westenberger et al., 2005) but this has not been supported by other analyses (de Freitas et al., 2006).

The *T. cruzi* (CL Brener strain; DTU TcIIe) haploid genome contains ∼12,000 genes and is approximately 55 Mb in size (El-Sayed et al., 2005), considerably larger than both *Trypanosoma brucei* (26 Mb) (Berriman et al., 2005) and *Leishmania major* (33 Mb) (Ivens et al., 2005). Evidence suggests the genomes of other *T. cruzi* strains are likely to differ from the CL Brener genome both structurally and in overall size. Pioneering flow cytometric studies showed that total DNA content varies significantly between *T. cruzi* strains, including between clones derived from the same source (Dvorak et al., 1982) and even between subclones derived from a cloned isolate (McDaniel and Dvorak, 1993). Complementary studies have revealed karyotypic diversity between strains, including often large differences in the size of homologous chromosomes (heteromorphy), thereby reinforcing the concept of genome plasticity and its potential importance with respect to the generation of genetic and phenotypic diversity (Engman et al., 1987; Henriksson et al., 1996, 2002; Dujardin et al., 2000; Brisse et al., 2003; Pedroso et al., 2003; Porcile et al., 2003; Vargas et al., 2004; Branche et al., 2006).

The phenomenon of genomic plasticity is also well documented in *T. brucei* (Gibson and Garside, 1991; Kanmogne et al., 1997; Melville et al., 1998, 2000; Hope et al., 1999; Callejas et al., 2006) and *Leishmania* spp. (Victoir et al., 1995; Britto et al., 1998; Inga et al., 1998; Kebede et al., 1999). Such genomic structural variation in trypanosomatids is thought to be generated predominantly by expansion and contraction of repetitive sequence arrays, but also by chromosomal fusion or fission events (reviewed by Dujardin et al., 2000). Hybridisation is a potential additional mechanism for the generation of karyotype variation and DNA content differences. For example, the presence of homologous chromosomes of two distinct size modes in members of DTUs TcIId and TcIIe is clearly a result of hybridisation (Henriksson et al., 2002; Brisse et al., 2003).

Experimental hybridisation experiments have shown that the capacity for genetic exchange has been retained by *T. cruzi* and also highlight the potential impact of hybridisation on DNA content (Gaunt et al., 2003). Gaunt et al. (2003) isolated hybrid *T. cruzi* clones after co-passage in vitro of two genetically distinct TcI ‘parents’ through mammalian cell culture. The genotypes of these hybrids were most consistent with a hybridisation mechanism that involves diploid–diploid fusion followed by genome erosion. This bears similarity to mating cycles in the pathogenic yeast *Candida albicans*, which undergoes tetraploid formation by diploid fusion followed by parasexual (non-meiotic) genome reduction in response to particular growth conditions (Hull et al., 2000; Magee and Magee, 2000; Bennett and Johnson, 2003). However, diploid fusion in *T. cruzi* is in contrast with the related pathogen *T. brucei*, for which laboratory crosses usually generate diploid progeny showing independent assortment of markers, which indicates that meiosis occurs (Gibson, 1989; Sternberg et al., 1989; Turner et al., 1990; MacLeod et al., 2005). *Trypanosoma brucei* crosses also generate polyploid progeny relatively frequently; but this appears to depend on which parental strains are used, and has been ascribed to fusion of unreduced gametes (Gibson et al., 1992, 2008; Gibson and Bailey, 1994; Hope et al., 1999). Meiosis-specific genes have been identified in the genomes of both *T. brucei* and *T. cruzi* (El-Sayed et al., 2005; Ramesh et al., 2005) raising the possibility that meiosis also operates in *T. cruzi*. Furthermore, it is not known whether the diploid fusion – genome erosion model is relevant to natural populations of *T. cruzi*, for example, hybrid DTUs TcIId and TcIIe.

We investigated the relationship between hybridisation and DNA content in *T. cruzi* by analysing natural and experimental hybrids and their respective (putative) parents. By using flow cytometric analysis in conjunction with microsatellite genotyping it was possible to analyse the relative DNA content and to estimate the ploidy of a large panel of clones. Furthermore it was possible to monitor changes in DNA content in the experimental hybrids under various growth conditions. The results show that the laboratory-generated *T. cruzi* hybrids described by Gaunt et al. (2003) are relatively stable sub-tetraploid organisms, whereas natural hybrid populations (TcIId and IIe) are largely diploid. We discuss the results in the context of recent advances in the understanding of *T. cruzi* genetic subdivisions and the mechanism of hybridisation.

## 2 Materials and methods

### 2.1 Parasite stocks and cloning procedure

A panel of 54 *T. cruzi* clones derived from wild type isolates and representing all six DTUs was assembled (Table 1). In addition, six TcI hybrid clones generated in vitro (Gaunt et al., 2003) were used in some experiments. Unless otherwise stated parasites were cultivated in supplemented RPMI liquid medium at 28 °C as previously described (Carrasco et al., 1996). Isolates that had not been previously cloned were cloned as described previously (Yeo et al., 2007). Briefly, 10^3^ parasites were mixed with 2.4 ml 0.9% NaCl (w/v) and 0.6 ml molten 3% (w/v) low melting point (LMP) agar, the mixture was then poured onto solid blood agar plates, allowed to set and incubated at 28 °C for 3–6 weeks. Once colonies became visible they were picked using a sterile pipette tip and inoculated into liquid culture.

### 2.2 Flow cytometry analysis

Approximately 1 × 10^7^ *T. cruzi* cells were taken from mid-log phase liquid cultures and centrifuged at 800*g* for 10 min at 4 °C. The supernatant was aspirated and the pellet gently re-suspended in ice-cold PBS, centrifuged again and the final cell pellet re-suspended in 300 μl PBS. Ice-cold 100% methanol (700 μl) was added and the tube was gently inverted several times. The cell suspension was placed on ice for 10 min before incubation at 4 °C for up to 48 h. Fixed cells were centrifuged at 200*g* for 10 min at 4 °C and the supernatant discarded. The pellet was washed in 5 ml cold PBS, re-sedimented and washed a second time. The cells were finally re-suspended in 1 ml PBS and samples were prepared for flow cytometry by diluting to a final density of 1 × 10^6^ cells/ml with PBS. Propidium iodide and RNAse A were both added to final concentrations of 10 μg/ml and samples were incubated for 45 min at 37 °C protected from light.

Fluorescence was detected using FACScan or FACSCalibur flow cytometers on channels FL3 or FL2, respectively. A minimum of 10,000 events were counted for each sample and each sample was run at least three times. FlowJo software (Tree Star Inc., Oregon, USA) was used for data analysis. After gating out debris and cell clumps the data were plotted as FL2 or FL3 area histograms. Gates were created for G1-0 (2*n*) peaks and for G2-M (4*n*) peaks. Mean G1-0 values were taken to infer relative DNA content. The coefficient of variation (CV) was recorded for each fluorescence peak. Each strain was tested in triplicate at a minimum and a control *T. cruzi* strain, Esm cl3, was included in every run as an internal standard. Relative DNA content values were calculated as a ratio compared with the internal standard. For experimental hybrids, the ratios relative to each parent (PI or PII) were also recorded using the mean Esm:PI and Esm:PII ratios from 12 independent experiments as standard values for conversions to parent:hybrid ratios.

### 2.3 Microsatellite analysis

All 54 cloned strains were genotyped at eight microsatellite loci: MCLF10 (Oliveira et al., 1998), 10101(TA), 6925(TG)a, 11283(TCG), 7093(TC), 10101(TC), 6925(CT) and 7093(TCC) (Supplementary Table S1). The latter seven loci were selected from a recently developed panel of new di- and tri-nucleotide repeat loci (Llewellyn et al., 2009) based on their utility in typing all the genetic subdivisions of *T. cruzi*. For each primer pair the forward sequence primer was labelled with one of four fluorescent labels with distinct emission spectra. The four dyes were 6-FAM, TET (Proligo, Germany), NED and VIC (Applied Biosystems, UK).

A standard 10 μl PCR reaction containing 2 ng DNA, 4 mM MgCl_2_, 34 μM dNTPs, 0.75 pmols of each primer and 1 U of *Taq* polymerase (Bioline) was used to amplify all loci. Amplification conditions were as follows: an initial denaturing step of 5 min at 95 °C followed by 30 amplification cycles (20 s at 95 °C, 20 s at 57 °C, 20 s at 72 °C) and a final elongation step of 10 min at 72 °C.

For analysis of fragment lengths, 0.5 μl from each reaction sample was added to a well of a 96 well sequence analysis plate containing 9.5 μl de-ionised formamide and 0.025 μl of G500 LIZ size standard (Applied Biosystems, UK). Samples were analysed using a 48-capillary 3730 DNA analyser (Applied Biosystems, UK). The data generated were analysed using Genemapper v3.5 software (Applied Biosystems, UK). The size of different PCR products (alleles), visualised as fluorescence peaks, were determined automatically by the software using the size standard to calibrate the calculations. All allele size calls made by the software were checked manually. In cases of suspected confounding effects due to artefactual stutter patterns and/or 3′-A additions, reactions were repeated until a consistent genotype was identified. Similarly, any samples showing evidence of >2 alleles per locus were subjected to authentication by technical replicates. The microsatellite genotypes were used to infer a measure of genetic distance between all possible pairs of strains (pairwise distance [*D*_AS_]) under the assumptions of the infinite-alleles model (IAM) (Kimura and Crow, 1964) by calculating 1 − *s*/*n*, where *s* is the total number of shared alleles across all loci and *n* is the number of loci, using the programme MICROSAT v1.5d (http://hpgl.stanford.edu/projects/microsat/).

### 2.4 Passage through severe combined immunodeficient (SCID) mice

Cultures of parental (PI and PII) and hybrid (2A2, 2D9, 2F9, 2C1, 1C2, 1D12) *T. cruzi* clones were maintained until late stationary phase in liquid growth medium. Aliquots (5 ml) were taken and the parasites were centrifuged at 800*g* for 5 min, the supernatant was discarded and the cell pellet was re-suspended in 1 ml of sterile saline solution (0.9% NaCl (w/v)). The number of metacyclic trypomastigotes was determined after fixing an aliquot of the parasites with an equal volume of 4% (w/v) paraformaldehyde in PBS.

For each strain, a 200 μl inoculum of sterile saline solution containing a total of 30,000 metacyclic trypanosomes and one control inoculum (saline only) were prepared and used for peritoneal inoculation of 4 week old male SCID mice. Parasitaemias were monitored every 3–4 days by phase microscopy of a drop of tail blood. Upon observation of elevated parasitaemia approximately 100 μl of tail blood was taken to inoculate normal in vitro cultures. Once cultures had entered log-phase growth, samples were taken and DNA content was measured as described. Cultures were maintained for up to 8 weeks; if additional samples were required fresh cultures were inoculated from stabilates. All mouse work was performed in accordance with local regulations for animal experimentation.

### 2.5 Cold stress and heat shock

For one parental clone, PI and two experimental hybrid clones, 1C2 and 2D9, samples of 4 × 10^6^ cells, in a total volume of 2 ml, were incubated under the following conditions: (i) 4 °C for 24 h; (ii) 4 °C for 48 h; (iii) 4 °C for 72 h; (iv) 42 °C for 2 min; (v) 42 °C for 10 min; (vi) 44 °C for 2 min; (vii) 44 °C for 10 min; (viii) 47 °C for 2 min; (ix) 47 °C for 10 min; (x) 50 °C for 30 s; (xi) 50 °C for 2 min; (xii) 50 °C for 10 min. The samples were then returned to normal growth conditions and were assessed microscopically for cell growth and viability every 2–3 days. After 2 weeks it was determined that incubation at 47 °C for 10 min was the most severe growth condition that had still permitted recovery of viable cultures (see Results). Cultures corresponding to this condition were maintained for up to 8 weeks in log-phase growth by re-passage into fresh media every 7–10 days and samples were processed for flow cytometric analysis to assess changes in DNA content as described above.

### 2.6 Nutritional stress

For nutritional stress, aliquots of normal growth media were diluted to varying degrees with PBS. The dilutions used were as follows (%RPMI media/%PBS, v/v) 100/0, 90/10, 75/25, 65/35, 50/50, 34/66, 20/80, 10/90, 1/99, 0.1/99.9. Ten 1.5 ml cultures, one at each dilution, of the parental clone, PI, and of two hybrid clones, 1C2 and 2D9, were prepared to a final density of 1 × 10^6^ cells/ml, in 24 well culture plates. The cultures were incubated under normal growth conditions. After 1 week all the cultures were sub-passaged (dilution factor = 1:10) to fresh plates in media identical to the primary cultures. After a further week the cells from each culture were harvested by centrifugation at 800*g* for 10 min and the pellets were re-suspended in normal liquid growth media. The cultures were assessed microscopically for cell growth and viability every 2–3 days. It was determined that the growth media containing 1% RPMI/99% PBS was the most severe nutritional stress condition that had still permitted recovery of viable organisms (see Results). Once the cultures corresponding to this growth condition had recovered to log-phase growth they were maintained for up to 8 weeks by re-passage to fresh media every 7–10 days. Flow cytometric analysis was carried out as described above in order to assess changes in DNA content.

### 2.7 Statistics

To test for correlation between genetic distance and differences in relative DNA content a Mantel test (Mantel, 1967) was performed with 9,999 permutations, using the programme GenAlex (Peakall and Smouse, 2006). Student’s *t*-tests and ANOVA with either Tukey’s Honestly Significant Differences (HSD) or Games–Howell multiple comparison post-hoc tests were performed using SPSS v.14 (SPSS Inc., USA).

## 3 Results

### 3.1 DNA content of natural T. cruzi populations

The DNA content of 54 *T. cruzi* clones was measured using flow cytometry of fixed parasites stained with the fluorescent DNA-binding dye propidium iodide. All strains gave traces with readily identifiable G1-0 (2*n*) and G2-M (4*n*) peaks, and the ratio between these peaks was typically in the range of 1.95–2.05, indicating a linear relationship between fluorescence intensity and DNA content. No haploid sized peaks were observed in any sample. We did not attempt to measure the relative contribution of the nucleus and kinetoplast but kDNA is normally 20–25% of the total DNA content (de Souza, 2003). CV values for G1-0 peaks were typically between 4% and 9%. This is comparable to previous results for *T. cruzi* (Dvorak et al., 1982) and other unicellular eukaryotes (e.g. yeasts) subjected to equivalent analyses (Haase and Reed, 2002).

As a species overall, *T. cruzi* displayed almost two-fold variation in relative DNA content (control:test ratio) ranging from 0.755 for strain C8 cl1 (TcI) to 1.438 for strain Tu18 cl2 (TcIIb). There were clear and often large differences in relative DNA content within and between DTUs (see following sections). Under the assumption that the relative contribution of nuclear and kinetoplast DNA to G1-0 peaks was equivalent between strains, the relative DNA content ratio values were converted to estimated genome sizes in megabases by calibration with the estimated total genome size of CL Brener (106.4–110.7 Mb) (El-Sayed et al., 2005) (Table 2). According to these estimates some *T. cruzi* strains contain up to ∼70 Mb more DNA than others.

### 3.2 Intra-DTU variation

**Fig. 1:**
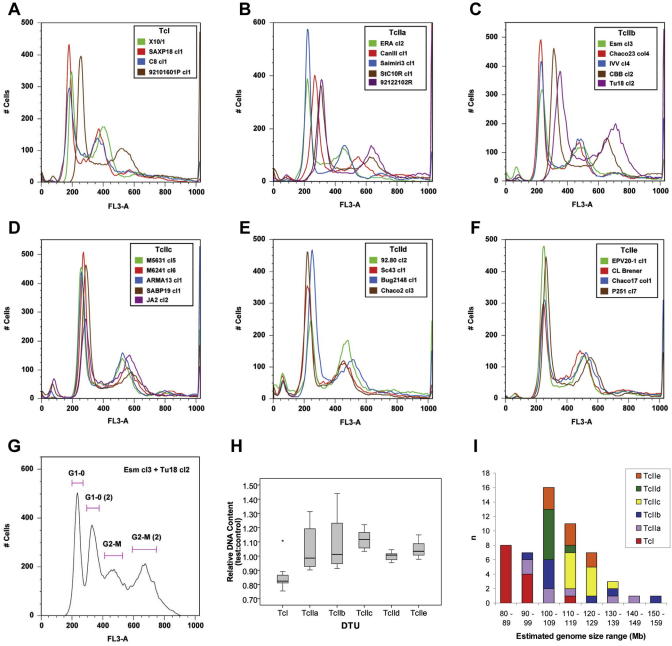
Flow cytometric analysis of relative DNA content in *Trypanosoma cruzi* discrete typing units (DTUs). (A–F) Overlaid DNA histograms for multiple cloned *T. cruzi* strains illustrating levels of variation within different DTUs. (G) DNA histogram for mixed sample population of two TcIIb strains with striking DNA content differences. (H) Box plot summary of variation within and between DTUs; grey boxes, inter-quartile ranges; horizontal lines inside grey boxes, median values; upper and low whiskers are the largest and smallest non-outlying values, respectively; asterisk, outlying value; outlying data were determined by the statistical software (SPSS v.14), additional putative outliers were identified (see Section 3.2). (I) Chart showing spread of estimated genome sizes for all samples across 10 Mb size categories; the number of strains from each DTU in each category is indicated by the split shading of the bars.

A box plot (Fig. 1H) illustrates the differences between *T. cruzi* DTUs. TcI, TcIIc, TcIId and TcIIe were relatively homogenous in terms of DNA content compared with TcIIa and TcIIb. This is illustrated by the DTU-specific inter-quartile ranges (IQRs), which were much larger for TcIIa and TcIIb and also by overlaying DNA histograms, which revealed the wider variation in G1-0 peak positions for TcIIa and TcIIb (Fig. 1A–F). To verify differences in DNA content between independent samples, mixtures of pairs of strains were analysed using equal numbers of cells from each sample. For sample pairs that had relatively small differences in DNA content the resulting G1-0 peak had increased CVs (i.e. peak width) compared with the histograms for each sample analysed alone (data not shown). For sample pairs with larger differences in DNA content, for example Esm cl3 and Tu18 cl2, distinct G1-0 and G2-M peaks could be distinguished for each isolate (e.g. Fig. 1G).

In addition to overall variability within groups, it was clear that some groups contained strains with DNA contents raised above the apparent normal range for their DTU. This can be visualised in the overlaid histograms as G1-0 peaks that are shifted to the right along the *x*-axis. In particular we suspected that the presence of five strains in the dataset might be misleading in terms of the typical DNA content of their DTUs. Within TcI, strain 92101601P cl1 had a DNA content 30.4% higher than the TcI mean; within TcIIa, strains 92122102R and StC10R cl1 had DNA contents 23.5% and 21.7% higher than the TcIIa mean, respectively; and within TcIIb, strains CBB cl2 and Tu18 cl2 had DNA contents raised by 19.7% and 31.9%, respectively. Besides these strains the next largest increases in DNA content from the means were 3.4% (TcI), 2.6% (TcIIa) and 6% (TcIIb). The remaining DTUs are characterised by relative homogeneity between strains, the maximum increases from the mean being 9% (TcIIc), 4.5% (TcIId) and 9.6% (TcIIe). Furthermore, strains 92101601P cl1, 92122102R and StC10R cl1 originate from North America and may therefore be genetically distinct from other (South American) strains in the DTU to which they have been assigned. The five strains mentioned were therefore designated as potential outliers and the subsequent ANOVA tests were performed both with and without these strains.

### 3.3 Inter-DTU variation

Superficial comparison of DTUs suggested that there were differences between them; comparisons of the mean relative DNA content of each DTU showed that they could be ordered as follows TcIIc > TcIIb > TcIIa > TcIIe > TcIId > TcI (outliers included) or TcIIc > TcIIe > TcIId > TcIIb > TcIIa > TcI (outliers excluded). To test whether differences between DTUs were significant, ANOVA was applied to the data. The result of the ANOVA test was a value of *F* = 8.476 (*P* < 0.001), thus the null hypothesis that average relative DNA content values are equal between DTUs was strongly rejected. A more informative analysis was carried out in the form of multiple pairwise comparisons between each DTU by ANOVA. These tests indicated that as a group, TcI had a significantly lower relative DNA content than TcIIc, TcIId and TcIIe and that TcIIc had a significantly higher relative DNA content than TcIId. All other pairwise comparisons were not significant; *P*-values for all pairwise comparisons are given in Table 3. Finally, a pairwise ANOVA was performed between TcI and all other DTUs combined (i.e. ‘TcII’) and this showed that the average DNA content of ‘TcII’ was significantly larger than that of TcI (*F* = 35.907, *P* < 0.001).

To test whether the presence of the outlying samples could have affected pairwise comparisons, the analyses were performed with the previously identified potential outliers removed from the dataset (92101601P cl1, 92122102R, StC10R cl1, Tu18 cl2 and CBB cl2). The overall ANOVA statistic *F* was 28.948 (*P* < 0.001) and the pairwise comparisons showed additional significant differences between DTUs (Table 3). In particular, TcI had a lower DNA content compared to all other DTUs and TcIIc had a higher DNA content than all other DTUs except TcIIe.

When the DNA contents of natural hybrid populations (DTUs TcIId and TcIIe) were compared with the parental populations (TcIIb and TcIIc) it was apparent that the values for the hybrids fit within the lower range of values observed for the parental DTUs, thus indicating a broadly equivalent ploidy level for these natural hybrids and their parents. Therefore, although aneuploidy is a feature of strain CL Brener (TcIIe) (Obado et al., 2005; Branche et al., 2006), our results suggest that the level of aneuploidy in hybrid DTU genomes is likely to be relatively limited.

### 3.4 Microsatellite analysis

**Fig. 2:**
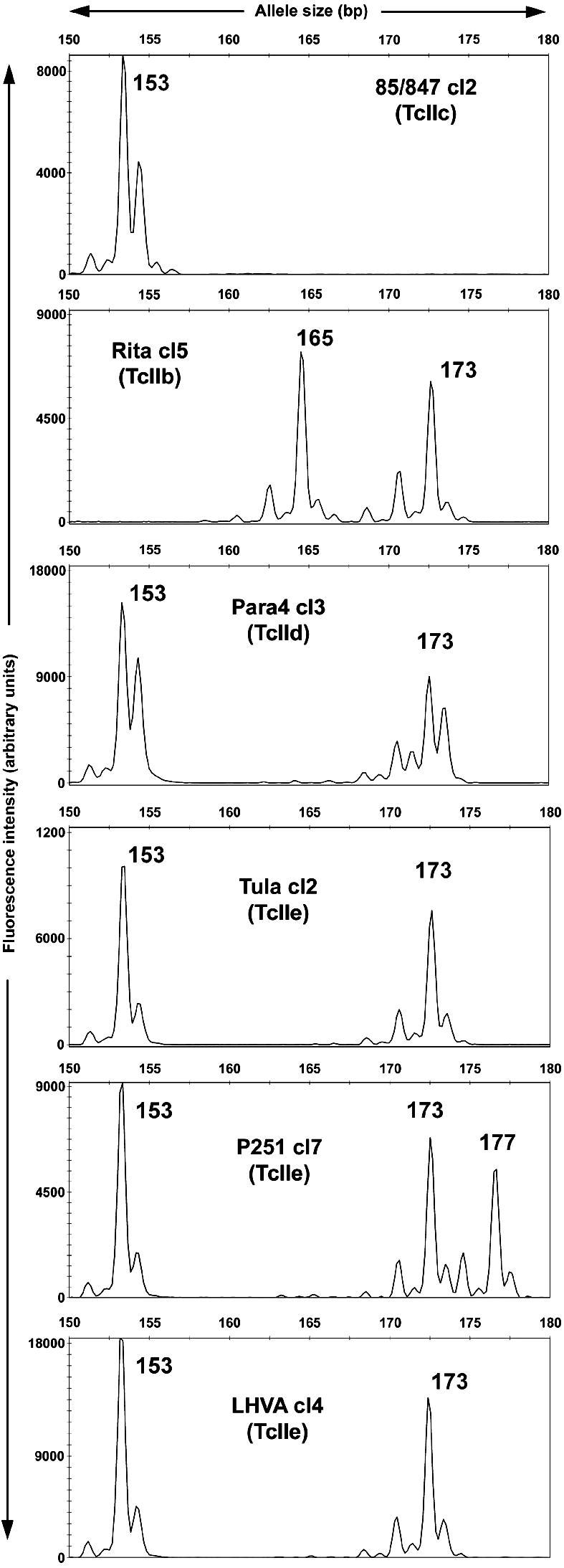
Genotyping of microsatellite locus 10101(TA). The *x*-axis shows PCR product (allele) size; *y*-axis indicates fluorescence intensity (arbitrary units). Each panel shows the result from a single sample as indicated. Note the presence of three alleles in P251 cl7 and the presence of both TcIIb and TcIIc alleles in TcIId/IIe hybrid samples. Allele sizes are shown adjacent to corresponding peaks.

All 54 cloned strains were genotyped at eight microsatellite loci (Table 4). As in previous studies of microsatellite variation in *T. cruzi* (Oliveira et al., 1998; Valadares et al., 2008), the vast majority of genotypes consisted of only one or two allele sizes. In fact, only a single genotype consisted of >2 allele sizes: strain P251 cl7 (TcIIe) reproducibly presented three distinct product sizes at locus 10101(TA) (Fig. 2), indicating that it is minimally trisomic at this locus. A number of DTU-specific alleles were identified (see Table 4). As for other markers (Machado and Ayala, 2001; Westenberger et al., 2005), heterozygosity was a clear feature for all strains from TcIIe (8/8 loci, P251 cl7 excluded) and TcIId (seven strains at 8/8 loci; one strain at 7/8 loci). Many of the microsatellite alleles identified in TcIId and/or TcIIe strains were also present in TcIIb or TcIIc strains (e.g. 10101(TA), Fig. 2). In most cases this is likely a result of the hybridisation event(s) between TcIIb and TcIIc that gave rise to TcIId and TcIIe and suggests that these alleles have not been subject to subsequent mutation events. Overall the microsatellite genotypes are consistent with the relative DNA content measurements and taken together they indicate that the hypothesis of diploidy (Gibson and Miles, 1986; Tibayrenc et al., 1986) applies generally to all *T. cruzi* DTUs, including hybrids, but with limited aneuploidy as a feature of some strains.

The microsatellite data were used to infer a measure of genetic distance (*D*_AS_) between strains. Across the sample, pairwise genetic distance was found to strongly correlate with pairwise difference in relative DNA content by a Mantel test (*r* = 0.365, *P* < 0.001). Correlation between independent genetic markers has been considered strong evidence of a clonal population structure for *T. cruzi* (Tibayrenc, 1999). The highly significant correlation between pairwise genetic distance and DNA content could therefore be seen as consistent with a model under which differences in DNA content are accrued gradually during independent clonal evolution of different strains. Nevertheless, many distantly related strains (e.g. CanIII cl1 and 85/847 cl2) had similar DNA contents and, more importantly, some pairs of strains with identical multilocus microsatellite genotypes presented large differences in DNA content (e.g. IVV cl4 and Tu18 cl2). This indicates that, in addition to a general trend of gradual diversification of genome size during independent clonal evolution, other factors may cause more rapid changes in the relative DNA content of different strains.

### 3.5 DNA content of experimental hybrids and their parents

**Fig. 3:**
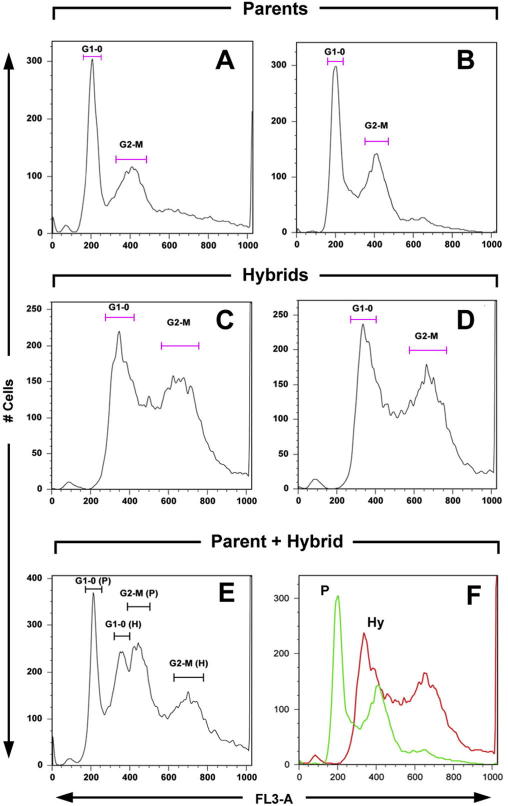
DNA histograms for experimental hybrid and parental clones. The *x*-axes represent fluorescence intensity (arbitrary units) and the *y*-axes represent number of events in each fluorescence channel. (A) parent PI; (B) parent PII; (C) hybrid 1C2; (D) hybrid 2F9; (E) mixed sample of parent PI and hybrid 2D9; (F) overlaid histograms of parent PII (P, green or light grey trace) and hybrid 2C1 (Hy, red or dark grey trace).

Measurements of the relative DNA contents of six laboratory generated hybrid TcI clones (Gaunt et al., 2003) and their parental clones demonstrated that the hybrids clearly had a higher DNA content than the parents as indicated by the position of the G1-0 peaks (Fig. 3). For the hybrids the standard DNA content measure (ratio compared to the control strain Esm cl3) was converted to a ratio versus each parent and then averaged to give an estimate of the ploidy of the hybrids. The mean parent:hybrid ratio varied from 1.65 for clone 2D9 to 1.72 for clone 2A2 (mean ratio across all hybrids = 1.69). These values are most consistent with the hybrids having an intermediate ploidy level between 3*n* and 4*n*; this represents further evidence that they have undergone limited genome reduction from a tetraploid fusion product as previously suggested (Gaunt et al., 2003). Differences between parents and hybrids were supported by analyses of mixtures of parent and hybrid samples, which allowed the identification of distinct parental and hybrid cell populations with DNA contents matching the respective single sample runs (e.g. Fig. 3E).

### 3.6 DNA content stability in sub-tetraploid hybrids

Given our results showing that naturally occurring *T. cruzi* hybrid DTUs are approximately diploid, and that the experimental sub-tetraploid hybrids may have an unstable DNA content, we predicted that the experimental hybrids might be capable of returning to a (diploid) DNA content equivalent to that of the parental strains. We investigated the possibility of rapid ploidy reductions associated with the following conditions: (i) passage through a mammalian host; (ii) temperature-induced stress; (iii) nutritional stress.

Positive infections of SCID mice were established for all six experimental hybrids and both parental clones. For all the infecting strains, bloodstream form (BSF) trypomastigotes were recovered from animals between 45 and 57 days p.i. and used to establish log-phase epimastigote cultures. Firstly, this demonstrates that these in vitro generated hybrids are capable of all the morphogenic transitions required to complete a full life cycle, and are able to survive in a mammalian host, albeit one that is immunocompromised. The recovered parasites were maintained in laboratory culture and the DNA content of each parental and hybrid population was assessed by flow cytometry. The results of the analysis showed marginal increases (∼2–3%) for both parents and one hybrid (2C1) and small decreases (∼2–6%) for the remaining five hybrids (Table 5). One of the reductions in DNA content (hybrid clone 2A2) was significant according to Student’s *t*-tests but all of the experimental hybrids remained sub-tetraploid. Thus, passage through a mammalian host was associated with only modest changes in DNA content rather than any dramatic ploidy shifts.

Initial tests were performed to establish stress condition parameters (see Materials and methods). For the time periods tested, incubation at 4 °C had no discernable effect on subsequent growth of recovered parasites. The most severe heat shock parameter that still permitted recovery of viable cultures was incubation at 47 °C for 10 min and the most severe nutritional stress condition was growth for 2 weeks in normal liquid growth media diluted to 1% with PBS. To test the stability of the DNA content of parental and hybrid organisms under these stress conditions, flow cytometric analysis was performed after the application of heat shock or nutritional stress followed by recovery of surviving organisms and re-establishment of log-phase growth (Table 5). For the one parent (PI) and two hybrid cell lines (2D9, 1C2) that were tested, DNA contents decreased by up to 5% after heat shock or 9% after nutritional stress. However, *t*-tests showed that only one of the reductions (PI, heat shocked) was statistically significant. This was also evident from examination of fluorescence histograms, which showed only minor differences in G1-0 peak position between samples before and after the stress experiments (not shown).

## 4 Discussion

We investigated DNA content variation in 54 *T. cruzi* strains and six experimentally generated hybrids in the context of genetic subdivision and hybridisation events. Clear and often large differences in DNA content were identified. The maximum difference observed between strains was 47.5%, slightly more than a previous study of 33 cloned isolates derived from six source stocks, among which relative DNA content varied by up to 41% (Dvorak et al., 1982). The availability of a reliable value for the CL Brener genome size (106.4–110.7 Mb) (El-Sayed et al., 2005) allowed us to estimate that the known DNA content of *T. cruzi* now ranges from ∼80 Mb up to ∼150 Mb, although >90% of strains contained less than 130 Mb. For these estimates we assumed that the proportion of nuclear and kinetoplast DNA is the same across strains but this might not always be the case. Separate measurements of the DNA in the different organelles will be required to resolve this. Genome size estimates for strains other than CL Brener have previously relied on densitometric analysis of karyotype gels, which generally lead to underestimations of total genome size due to technical limitations, chiefly, failure of all the genetic material in a sample to migrate into the gel and hence contribute to the size estimations.

We found that TcI strains typically have lower DNA contents than strains from other DTUs corroborating previous findings based on relatively small samples (Dvorak et al., 1982; Nozaki and Dvorak, 1991; Vargas et al., 2004; Branche et al., 2006). Karyotype comparisons have also indicated that TcI strains have fewer, and generally smaller, chromosomes than TcII strains (Pedroso et al., 2003; Vargas et al., 2004). While TcI had the lowest mean DNA content, TcIIc was found to have the highest. TcIIc has been proposed as a distinct ancestral *T. cruzi* lineage (de Freitas et al., 2006) or alternatively as a hybrid lineage formed by hybridisation between TcI and TcIIb (Westenberger et al., 2005). Either way, the available evidence indicates that TcIIc has evolved in isolation from other DTUs for a time sufficient for the evolution of DTU-specific multilocus enzyme electrophoresis (MLEE) profiles (Brisse et al., 2000), single nucleotide polymorphisms (SNPs) (Machado and Ayala, 2001; Westenberger et al., 2005), distinct niche-associations (Yeo et al., 2005) and, as shown in this study, DTU-specific microsatellite alleles and a characteristic genome size.

There was a significant correlation between relative DNA content and genetic distance, perhaps best exemplified by the distinct differences in mean estimated genome sizes of ancestral *T. cruzi* lineages, TcI (88.4 Mb), TcIIb (106.5 Mb) and TcIIc (119.2 Mb) (outliers excluded), which are separated by millions of years of evolution (Machado and Ayala, 2001). Karyotype variability also correlates with genetic subdivisions (Henriksson et al., 2002; Brisse et al., 2003; Pedroso et al., 2003) and so it seems likely that structural changes to the genome accrue gradually during clonal diversification of independent lineages. Multiple mechanisms are likely to be involved but the primary process is likely to be expansion and contraction of tandem repeat arrays (Dujardin et al., 2000). Repetitive sequences make up approximately 50% of the CL Brener genome (El-Sayed et al., 2005) and so it would be interesting to see if the smaller TcI genome had such a high proportion. In fact, Vargas et al. (2004) reported that some repeat types are more abundant in TcII strains than TcI strains. Differential expansion of repeat arrays could also partly explain why TcII strains tend to have a higher proportion of larger chromosomes (Vargas et al., 2004; Branche et al., 2006), although in some instances this may be due to fusion of smaller chromosomes, which would not contribute to changes in overall genome size.

Rather than invoking a model of genome size divergence akin to random drift there could be phenotypic differences between TcI and TcII that have resulted in different genome sizes. This idea gains tentative support from some existing evidence. Firstly, temperature-induced stress (growth at 35 °C) resulted in transient increases in DNA content of 3–11% in TcII strains but not in TcI strains, although much of the increase occurred in the kinetoplast (Nozaki and Dvorak, 1993). Second, TcII strains exhibited microsatellite instability when grown in the presence of hydrogen peroxide whereas TcI strains did not (Augusto-Pinto et al., 2003). Also, TcII strains may display higher intragenomic sequence diversity in some antigenic multigene families than TcI strains (Cerqueira et al., 2008). Such results have led to speculation that DNA mismatch repair (MMR) efficiency is lower in TcII than in TcI and this has resulted in higher genetic diversity that may be linked to differences in pathogenicity between the lineages (Machado et al., 2006). Further studies are clearly required but if some TcII lineages do share some mechanism that lessens restraints on the expansion of repetitive regions, this could be an explanation for the increased genome size compared with TcI.

The finding that some samples had large amounts of ‘extra’ DNA, equivalent to an estimated extra 15–30 Mb compared with the means for their DTUs, suggests additional mechanism(s) for more rapid changes in DNA content. This represents many chromosomes worth of genetic material, and can only reasonably be explained by duplication of chromosomal material either endogenously or through genetic exchange. These observations are reminiscent of those made for another TcIIb isolate (Y strain) for which different subclones were found to have DNA contents raised by 30% or 70% compared with the (cloned) source isolate (McDaniel and Dvorak, 1993). Dramatic changes in ploidy have also been documented in *Leishmania* spp. during in vitro cultivation (Dujardin et al., 2007) and also after genetic manipulation (Cruz et al., 1993; Mottram et al., 1996; Dumas et al., 1997; Martinez-Calvillo et al., 2005). It therefore appears that some trypanosomatids are capable of rapid and dramatic alteration of ploidy. In vitro cultivation of the *T. cruzi* strains used here, in the absence of most sources of selective pressure, may have increased the likelihood of such raised ploidy variants. Although the microsatellite profiles in this study were almost invariably mono- or bi-allelic, this would still be consistent with endogenous generation of aneuploidy/polyploidy since duplication of loci would not be detectable as new alleles.

To date, the only successful experimental cross of *T. cruzi* was between two TcI ‘parents’ and yielded six hybrid progeny clones (Gaunt et al., 2003). For the majority (10/11) of polymorphic microsatellite loci all parental alleles were present in the hybrids; however, at one microsatellite locus (L660) and also for one gene sequence (*tpn1*, tryparedoxin), parental alleles were absent. Thus, these hybrids appear to be aneuploid organisms originating from tetraploids that have undergone loss of genetic material to some degree. Measurement of relative DNA content/organism in this study has revealed the extent of this reduction. The hybrid organisms were found to have a mean DNA content 1.65–1.72 times greater than their parents. This is consistent with an aneuploid state between triploidy and tetraploidy, and assuming that tetraploid intermediates were produced, this corresponds to an approximate loss of 15.3% of the DNA from the tetraploid nucleus. The possibility remains that loss of parental alleles could have been brought about by gene conversion events although we consider this unlikely in view of the DNA content measurements. A quantitative analysis of copy number at multiple loci would permit a better estimation of the relative rates of physical allele loss as opposed to gene conversion events.

These hybrids had previously been maintained only as insect-associated life cycle stages. It therefore remained to be established whether these hybrids were viable in terms of their competence to complete a full life cycle. Furthermore, any potential (permanent) DNA reduction phenomena associated specifically with parasite life cycle stages found in mammalian hosts, or transitions involving such stages, would have been missed if only the insect forms were analysed. Transitions from tetraploidy towards diploidy have been observed in the model yeast *Saccharomyces cerevisiae*, as a result of both meiotic sporulation (Roman et al., 1955) and vegetative (i.e. mitotic) growth under both normal and environmentally stressful conditions, (Gerstein et al., 2006, 2008), and in the yeast pathogen *C. albicans*, in response to heat shock (Hilton et al., 1985) and growth under nutritional stress (Bennett and Johnson, 2003). Here, no such dramatic shifts in ploidy occurred. Small changes in DNA content were observed for *T. cruzi* parents and hybrids as a result of passage through a mammalian host, heat shock and nutritional stress, and although two of these changes were shown to be weakly significant, none were greater than the typical G1-0 peak CV (5–9%). This leads to the conclusion that the aneuploid DNA content of the hybrids is relatively stable, even under short-term stressful conditions. The possibility of further genome erosion during long-term growth in response to other conditions remains to be explored.

A degree of caution is required when interpreting the characteristics of these *T. cruzi* hybrids. In the earliest successful *T. brucei* laboratory crosses the hybrids were thought to be derived from unstable tetraploid intermediates produced by fusion of diploid parental cells (Paindavoine et al., 1986). In subsequent crosses most progeny were diploid but there has also been a significant frequency of hybrids with raised DNA contents (Paindavoine et al., 1986; Wells et al., 1987; Gibson et al., 1992, 1997, 2008; Gibson and Bailey, 1994; Hope et al., 1999). *Trypanosoma brucei* hybrid progeny with raised DNA content have been considered most likely to be triploids formed by fusions involving an unreduced gamete (i.e. 2*n* + *n*) (Gibson et al., 1992, 2008; Gibson and Bailey, 1994; Hope et al., 1999). Genetic analysis of large numbers of progeny clones has unequivocally shown that the mechanism of genetic exchange in *T. brucei* does involve meiosis (MacLeod et al., 2005), however, since haploid gamete stages have yet to be identified it has not been possible to determine whether the meiotic divisions occur before fusion i.e. classical meiosis, or after fusion to bring about a tetraploid to diploid reduction. Given these results and the shared ancestry of *T. brucei* and *T. cruzi* it appears premature to rule out the operation of meiosis in *T. cruzi*. The fact remains, however, that the genotypes of the experimental hybrids produced so far are not compatible with conventional meiosis.

Strains from hybrid DTUs TcIId/IIe were found to be homogenous in terms of DNA content and comparisons to the DNA contents of the TcIIb and TcIIc strains clearly showed that the hybrid organisms have a DNA content that is broadly equivalent to the two parental DTUs. Therefore, TcIId/IIe strains are expected to be approximately diploid. This was in keeping with the microsatellite analysis, which consistently showed that natural hybrid strains always had either one or two alleles per locus (with the exception of one trisomy), of which many could be identified as likely to be TcIIb- or TcIIc-derived. This presents a fundamental contrast to the multi-allelic microsatellite profiles (Gaunt et al., 2003) and the sub-tetraploid DNA contents of the experimental hybrids. The most obvious explanation is selective pressure against polyploids in the complex natural cycles of *T. cruzi*, which is not replicated in vitro. Diploidy of TcIId/IIe strains may therefore be related to their survival and success: they are widespread across the Southern cone region of South America and are found almost exclusively in domestic transmission cycles (Chapman et al., 1984; Miles et al., 1984; Barnabé et al., 2000, 2001a; Bosseno et al., 2002; Brenière et al., 2002; Virreira et al., 2006; Cardinal et al., 2008). A further important question is how the diploid state was reached. The hybridisation event(s) that created TcIId/IIe could have occurred through orthodox meiosis, diploid fusion followed by either meiotic reduction or parasexual reduction to the current diploid state, or other as yet unforeseen mechanisms.

If, in a putative tetraploid → diploid transition, losses of chromosomal homologues are random with respect to parentage then the resulting hybrids would be expected to inherit both alleles from the same parent (i.e. non-recombinant genotypes) at one third of all loci, whether the reduction mechanism involves meiotic divisions or parasexual genome erosion. Orthodox meiosis on the other hand would have resulted in F1 hybrids that received one chromosomal homologue from each parent and would therefore be expected to have recombinant genotypes at all loci. Subsequent rounds of selfing or backcrossing or gene conversion events would be expected to reduce heterozygosity. Strains from TcIId and TcIIe have recombinant genotypes at almost all loci so far examined, i.e. they have both TcIIb-like and TcIIc-like alleles (Robello et al., 2000; Machado and Ayala, 2001; Augusto-Pinto et al., 2003; Gaunt et al., 2003; Tran et al., 2003; Westenberger et al., 2005). At a minimum this suggests that the involvement of meiosis should be considered in future analyses of these hybridisation events. The *T. cruzi* genome contains genes encoding proteins specifically required for meiosis in other eukaryotes (e.g. *SPO11*, *DMC1*) (El-Sayed et al., 2005; Ramesh et al., 2005), although recent data shows that Spo11 can mediate non-meiotic genetic recombination (in the parasexual cycle of *C. albicans*) (Forche et al., 2008). Haploid *T. cruzi* forms have never been observed yet they would be required for orthodox meiosis.

Whether or not the fusion-reduction model observed in the laboratory is the sole mechanism of genetic exchange in *T. cruzi* thus remains unclear. A better understanding of the mechanism of reduction in the laboratory and resolving the finer details of known hybridisation event(s) in natural populations of *T. cruzi* would be facilitated by the capacity to efficiently generate larger numbers of experimental *T. cruzi* hybrids. Unfortunately an efficient in vitro mating system for *T. cruzi* remains elusive. If sexual reproduction in natural populations involves diploid → tetraploid → diploid cycles and/or non-Mendelian inheritance this would have important consequences for population genetic studies of the species and also provoke wider evolutionary questions.

## Figures and Tables

**Table 1:** *Trypanosoma cruzi* stocks used in the study.

Strain	Genotype (method)	Origin	Host/vector	Reference
X10/1	TcI (MLEE)	Belém, Brazil	*Homo sapiens*	Miles et al. (1978)
C8 cl1	TcI (MLEE)	La Paz, Bolivia	*Triatoma infestans*	Tibayrenc and Miles (1983)
PI (CJ007)	TcI (MLEE, RAPD)	Carajas, Brazil	*Didelphis marsupialis*	Carrasco et al. (1996)
PII (CJ005)	TcI (MLEE, RAPD)	Carajas, Brazil	Unidentified Bug	Carrasco et al. (1996)
B187 cl10^a^	TcI (rDNA, miniexon)	Pará state, Brazil	*Didephis marsupialis*	Yeo et al. (2007)
Chile C22 cl1^a^	TcI (MLEE)	Flor de Valle, Chile	*Triatoma spinolai*	Miles et al. (1984)
SAXP18 cl1^a^	TcI (MLEE)	Majes, Peru	*Homo sapiens*	Widmer et al. (1987)
JR cl4^a^	TcI (RAPD)	Anzoátegui, Venezuela	*Homo sapiens*	Carrasco (unpublished data)
92101601P cl1	TcI (MLEE, RAPD)	Georgia, USA	*Didelphis marsupialis*	Barnabé et al. (2001b)
Xe5740 cl1^a^	TcI (rDNA, miniexon)	Pará, Brazil	*Didephis marsupialis*	Llewellyn (unpublished data)
M13 cl2^a^	TcI (rDNA, miniexon)	Barinas, Venezuela	*Didelphis marsupialis*	Llewellyn (unpublished data)
M7 cl4^a^	TcI (rDNA, miniexon)	Barinas, Venezuela	*Didelphis marsupialis*	Llewellyn (unpublished data)
Xe1313 cl3^a^	TcI (rDNA, miniexon)	Carajas, Brazil	*Philander opossum*	Llewellyn (unpublished data)
CanIII cl1	TcIIa (MLEE)	Belém, Brazil	*Homo sapiens*	Miles et al. (1978)
StC10R cl1	TcIIa (MLEE, RAPD)	Georgia, USA	*Procyon lotor*	Barnabé et al. (2001b)
10 R26	TcIIa (MLEE)	Santa Cruz, Bolivia	*Aotus sp.*	Tibayrenc and Ayala (1988)
X10610 cl5^a^	TcIIa (rDNA, miniexon)	Guárico, Venezuela	*Homo sapiens*	Yeo et al. (2007)
92122102R	TcIIa (rDNA)	Georgia, USA	*Procyon lotor*	Sturm et al. (2003)
Saimiri3 cl1^a^	TcIIa (MLEE, RAPD)	Venezuela	*Saimiri sciureus*	Brisse et al. (2000)
ERA cl2^a^	TcIIa (rDNA, miniexon)	Anzoátegui, Venezuela	*Homo sapiens*	Yeo et al. (2007)
Esm cl3	TcIIb (MLEE)	São Felipe, Brazil	*Homo sapiens*	Miles et al. (1977)
Pot7a cl1^a^	TcIIb (rDNA, miniexon)	San Martin, Paraguay	*Triatoma infestans*	Yeo et al. (2007)
Pot7b cl5^a^	TcIIb (rDNA, miniexon)	San Martin, Paraguay	*Triatoma infestans*	Yeo et al. (2007)
Rita cl5^a^	TcIIb (MLEE)	São Felipe, Brazil	*Homo sapiens*	Miles et al. (1977)
Tu18 cl2	TcIIb (MLEE, RAPD)	Tupiza, Bolivia	*Triatoma infestans*	Brenière et al. (1998)
CBB cl2	TcIIb (MLEE)	Tulahuen, Chile	*Homo sapiens*	Brenière et al. (1991)
IVV cl4	TcIIb (MLEE, RAPD)	Cuncumen, Chile	*Homo sapiens*	Brenière et al. (1998)
Chaco23 col4	TcIIb (rDNA, miniexon)	Chaco, Paraguay	*Triatoma infestans*	Yeo (unpublished data)
M5631 cl5	TcIIc (MLEE)	Marajo, Brazil	*Dasypus novemcinctus*	Miles et al. (1981)
M6421 cl6	TcIIc (MLEE)	Belém, Brazil	*Homo sapiens*	Tibayrenc and Ayala (1988)
X9/3	TcIIc (MLEE)	Makthlawaiya, Paraguay	*Canis familiaris*	Chapman et al. (1984)
X109/2	TcIIc (MLEE)	Makthlawaiya, Paraguay	*Canis familiaris*	Chapman et al. (1984)
JA2 cl2^a^	TcIIc (MLEE)	Amazonas, Brazil	*Monodelphis sp.*	Miles (unpublished data)
ARMA13 cl1^a^	TcIIc (rDNA, miniexon)	Campo Lorro, Paraguay	*Dasypus novemcinctus*	Yeo et al. (2007)
ARMA18 cl3^a^	TcIIc (rDNA, miniexon)	Campo Lorro, Paraguay	*Dasypus novemcinctus*	Yeo et al. (2007)
CM25 cl2^a^	TcIIc (MLEE, RAPD)	Carimaga, Colombia	*Dasyprocta fugilinosa*	Brisse et al. (2000)
85/847 cl2^a^	TcIIc (rDNA)	Alto Beni, Bolivia	*Dasypus novemcinctus*	Brisse et al. (2001)
SABP19 cl1^a^	TcIIc (MLEE)	Vitor, Peru	*Triatoma infestans*	Miles (unpublished data)
Sc43 cl1	TcIId (MLEE)	Santa Cruz, Bolivia	*Triatoma infestans*	Tibayrenc and Miles (1983)
92.80 cl2	TcIId (MLEE)	Santa Cruz, Bolivia	*Homo sapiens*	Tibayrenc and Miles (1983)
Para4 cl3^a^	TcIId (rDNA, miniexon)	Paraguari, Paraguay	*Triatoma infestans*	Yeo et al. (2007)
Para6 cl4^a^	TcIId (rDNA, miniexon)	Paraguari, Paraguay	*Triatoma infestans*	Yeo et al. (2007)
Chaco2 cl3^a^	TcIId (rDNA, miniexon)	Chaco, Paraguay	*Triatoma infestans*	Yeo et al. (2007)
Vinch101 cl1	TcIId (MLEE)	Limari, Chile	*Triatoma infestans*	Widmer et al. (1987)
Bug 2148 cl1	TcIId (rDNA)	Rio Grande do Sul, Brazil	*Triatoma infestans*	Souto et al. (1996)
PAH179 cl5^a^	TcIId (MLEE)	Chaco, Argentina	*Homo sapiens*	Diosque (unpublished data)
CL Brener	TcIIe (MLEE, RAPD)	Rio Grande do Sul, Brazil	*Triatoma infestans*	Brisse et al. (1998)
Chaco17 col1	TcIIe (MLEE)	Chaco, Paraguay	*Triatoma infestans*	Yeo (unpublished data)
Chaco9 col15	TcIIe (MLEE)	Chaco, Paraguay	*Triatoma infestans*	Yeo (unpublished data)
Tula cl2	TcIIe (MLEE)	Tulahuen, Chile	*Homo sapiens*	Tibayrenc and Ayala (1988)
P251 cl7^a^	TcIIe (MLEE)	Cochabamba, Bolivia	*Homo sapiens*	Machado and Ayala (2001)
EPV20-1 cl1^a^	TcIIe (MLEE)	Chaco, Argentina	*Triatoma infestans*	Diosque (unpublished data)
LHVA cl4^a^	TcIIe (MLEE)	Chaco, Argentina	*Triatoma infestans*	Diosque (unpublished data)
VFRA1 cl1^a^	TcIIe (MLEE)	Francia, Chile	*Triatoma infestans*	Barnabé et al. (2001a)

a Clones prepared in this study.

**Table 2:** Relative DNA contents and genome size estimates of *Trypanosoma cruzi* clones used in this study.

Discrete typing unit	Strain	Relative DNA content (ratio:control)	SEM	Mean estimated genome size (Mb)	95% confidence intervals
TcI	C8 cl1	0.755	0.012	80.64	(77.11–84.24)
TcI	SAXP18 cl1	0.769	0.005	82.10	(79.69–84.54)
TcI	Chile C22 cl1	0.806	0.022	86.03	(80.70–91.50)
TcI	X10/1	0.824	0.023	87.99	(82.41–93.72)
TcI	JR cl4	0.837	0.009	89.37	(86.09–92.72)
TcI	B187 cl10	0.856	0.016	91.41	(86.72–96.22)
TcI	PII (CJ005)	0.866	0.009	92.47	(88.15–96.88)
TcI	PI (CJ007)	0.885	0.012	94.49	(89.02–100.11)
TcI	92101601P cl1	1.107	0.008	118.19	(114.13–122.33)
TcI	Xe5740 cl1	0.811	0.030	86.59	(79.91–93.48)
TcI	M13 cl2	0.814	0.026	86.87	(80.78–93.14)
TcI	M7 cl4	0.817	0.026	87.22	(81.13–93.49)
TcI	Xe1313 cl3	0.891	0.042	95.17	(85.58–105.06)
TcIIa	92122102R	1.312	0.026	140.13	(130.45–150.08)
TcIIa	StC10R cl1	1.294	0.025	138.13	(128.83–147.69)
TcIIa	X10610 cl5	0.902	0.016	96.35	(91.43–101.40)
TcIIa	Saimiri3 cl1	0.911	0.021	97.23	(91.36–103.26)
TcIIa	ERA cl2	0.943	0.007	100.71	(97.36–104.11)
TcIIa	10R26	0.986	0.020	105.25	(99.11–111.55)
TcIIa	CanIII cl1	1.090	0.017	116.44	(110.40–122.63)
TcIIb	IVV cl4	0.913	0.036	97.45	(88.79–106.38)
TcIIb	Rita cl5	0.934	0.026	99.71	(92.84–106.77)
TcIIb	Chaco23 col4	0.958	0.007	102.30	(98.82–105.83)
TcIIb	Esm cl3	1.000	0.000	106.78	-
TcIIb	Pot7a cl1	1.022	0.027	109.15	(101.42–117.10)
TcIIb	Pot7b cl5	1.156	0.024	123.45	(115.29–131.85)
TcIIb	CBB cl2	1.306	0.015	139.43	(132.73–146.29)
TcIIb	Tu18 cl2	1.438	0.017	153.58	(145.47–161.90)
TcIIc	M5631 cl5	1.034	0.044	110.40	(98.90–122.27)
TcIIc	X9/3	1.046	0.025	111.71	(106.81–116.71)
TcIIc	CM25 cl2	1.057	0.017	112.83	(107.01–118.80)
TcIIc	85/847 cl2	1.080	0.016	115.28	(109.54–121.15)
TcIIc	ARMA18 cl3	1.106	0.026	118.07	(109.90–126.47)
TcIIc	M6241 cl6	1.126	0.021	120.29	(113.12–127.65)
TcIIc	ARMA13 cl1	1.136	0.021	121.30	(114.02–128.79)
TcIIc	X109/2	1.166	0.023	124.50	(119.23–129.88)
TcIIc	JA2 cl2	1.193	0.019	127.35	(120.11–134.78)
TcIIc	SABP19 cl1	1.220	0.015	130.26	(123.88–136.79)
TcIId	Sc43 cl1	0.953	0.003	101.79	(99.23–104.37)
TcIId	Para6 cl4	0.962	0.016	102.75	(97.49–108.15)
TcIId	Chaco2 cl3	0.987	0.016	105.44	(100.03–110.98)
TcIId	Vinch101 cl1	1.005	0.006	107.29	(103.94–110.69)
TcIId	92.80 cl2	1.008	0.011	107.66	(103.16–112.25)
TcIId	Para4 cl3	1.013	0.025	108.19	(100.86–115.73)
TcIId	PAH179 cl5	1.020	0.019	108.90	(102.66–115.29)
TcIId	Bug2148 cl1	1.044	0.014	111.51	(106.26–116.89)
TcIIe	Tula cl2	0.977	0.006	104.35	(101.08–107.67)
TcIIe	LHVA cl4	0.997	0.010	106.50	(102.30–110.78)
TcIIe	CL Brener	1.017	0.024	108.55	(101.41–115.89)
TcIIe	EPV20-1 cl1	1.030	0.020	110.03	(103.56–116.68)
TcIIe	VFRA1 cl1	1.037	0.020	110.73	(104.35–117.28)
TcIIe	Chaco17 col1	1.044	0.034	111.47	(101.93–121.30)
TcIIe	Chaco9 col15	1.130	0.004	120.71	(117.47–123.99)
TcIIe	P251 cl7	1.149	0.023	122.65	(114.78–130.75)

**Table 3:** ANOVA *P*-values for DNA content differences between *Trypanosoma cruzi* Discrete Typing Units (DTUs).

DTU	TcI	TcIIa	TcIIb	TcIIc	TcIId	TcIIe
TcI		0.001	<0.001	<0.001	<0.001	<0.001
TcIIa	0.120		0.954	<0.001	0.924	0.175
TcIIb	0.068	1.000		0.004	1.000	0.613
TcIIc	<0.001	0.964	0.999		0.002	0.163
TcIId	<0.001	0.922	0.761	0.002		0.576
TcIIe	<0.001	1.000	0.988	0.240	0.394	

Lower triangle = All strains (*n* = 54), Games–Howell post-hoc test. Upper triangle = Outliers removed (*n* = 49), Tukey’s Honestly Significant Differences (HSD) post-hoc test.

**Table 4:** Microsatellite genotypes of *Trypanosoma cruzi* clones used in this study.

Highlighting indicates Discrete Typing Unit (DTU)-specific alleles (TcIId/IIe excluded).

**Table 5:** Relative DNA contents of experimental hybrids used in this study.

Condition	Clone	Mean control:test ratio	S.E.M.	Equivalent parent:hybrid ratio	% change	*t*-test *P*-value
	PI	0.873^a^	0.010	–	–	–

Wild type parents	PII	0.851^a^	0.010	–	–	–
In vitro generated hybrids	2A2	1.484	0.026	1.722	–	–
	2D9	1.421	0.040	1.649	–	–
	2F9	1.465	0.038	1.700	–	–
	2C1	1.446	0.026	1.678	–	–
	1C2	1.461	0.023	1.696	–	–
	1D12	1.481	0.015	1.719	–	–

Passage through SCID mouse	PI	0.876	0.017	–	+1.956	0.895
	PII	0.852	0.006	–	+3.071	0.949
	2A2	1.392	0.027	1.616	−6.161	0.049
	2D9	1.377	0.011	1.598	−3.073	0.178
	2F9	1.437	0.064	1.668	−1.916	0.737
	2C1	1.488	0.036	1.727	+2.916	0.378
	1C2	1.423	0.034	1.651	−2.624	0.475
	1D12	1.406	0.066	1.632	−5.104	0.304

Heat Shock	PI	0.823	0.002	–	−4.154	0.031
	2D9	1.364	0.034	1.583	−4.011	0.319
	1C2	1.386	0.034	1.609	−5.120	0.119

Nutritional stress	PI	0.838	0.008	–	−2.410	0.077
	2D9	1.291	0.035	1.499	−9.099	0.068
	1C2	1.391	0.024	1.614	−4.809	0.074

a Values differed slightly compared with separate described experiments (Table 2).
