# Supplementary material for: Flow cytometric analysis and microsatellite genotyping reveal extensive DNA content variation in Trypanosoma cruzi populations and expose contrasts between natural and experimental hybrids
Source: Int J Parasitol. 2009 Oct;39(12):1305–17. doi: 10.1016/j.ijpara.2009.04.001 (PMC2731025; doi:10.1016/j.ijpara.2009.04.001)
Supplement: Supplementary Table [file mmc1.doc]

Supplementary Table S1. Details of microsatellite loci used in the study

| **Primer code** | **Contig ID ^a^** | **Repeat type** | **Forward/Reverse Primer (5'-3')** |
| --- | --- | --- | --- |
|  |  |  |  |
| 6925(TG)a | 1047053506925 | (TG)n | TCGTTCTCTTTACGCTTGCA |
|  |  |  | TAGCAGCACCAAACAAAACG |
|  |  |  |  |
| 6925(CT) | 1047053506925 | (CT)n | CATCAAGGAAAAACGGAGGA |
|  |  |  | CGGTACCACCTCAAGGAAAG |
|  |  |  |  |
| 7093(TC) | 1047053507093 | (TC)n | CCAACATTCAACAAGGGAAA |
|  |  |  | GCATGAATATTGCCGGATCT |
|  |  |  |  |
| 7093(TCC) | 1047053507093 | (TCC)n | AGACGTTCATATTCGCAGCC |
|  |  |  | AGCCACATCCACATTTCCTC |
|  |  |  |  |
| 10101(TC) | 1047053510101 | (TC)n | CGTACGACGTGGACACAAAC |
|  |  |  | ACAAGTGGGTGAGCCAAAAG |
|  |  |  |  |
| 10101(TA) | 1047053510101 | (TA)n | AACCCGCGCAGATACATTAG |
|  |  |  | TTCATTTGCAGCAACACACA |
|  |  |  |  |
| 11283(TCG) | 1047053511283 | (TCG)n | ACCACCAGGAGGACATGAAG |
|  |  |  | TGTACACGGAACAGCGAAG |
|  |  |  |  |
| MCLF10 ^b^ | Unknown | (CA)n | GCGTAGCGATTCATTTCC |
|  |  |  | ATCCGCTACCACTATCCAC |
|  |  |  |  |

^a^ Refers to CL Brener genome sequence fragment (www.tigr.org).

^b^ Published in Oliveira et al. (1998).
